# Thrombolytic Therapy for Massive Pulmonary Embolism in an Antiphospholipid Syndrome Patient With Severe Thrombocytopenia: A Case Report

**DOI:** 10.1002/ccr3.71395

**Published:** 2025-11-18

**Authors:** Arman Ahmadzadeh, Samad Nazar Poor, Reza Hamneshin Behbahani, Pegah Soltani, Arian Tavasol, Mehdi Sheibani

**Affiliations:** ^1^ Department of Rheumatology, Loghman Hakim Hospital Shahid Beheshti University of Medical Sciences Tehran Iran; ^2^ Department of Adult Rheumatology, School of Medicine, Loghman Hakim Hospital Shahid Beheshti University of Medical Sciences Tehran Iran; ^3^ School of Medicine, Loghman Hakim Hospital Shahid Beheshti University of Medical Sciences Tehran Iran; ^4^ Student Research Committee, School of Medicine, Loghman Hakim Hospital Shahid Beheshti University of Medical Sciences Tehran Iran; ^5^ Student Research Committee, Faculty of Medicine Shahid Beheshti University of Medical Sciences Tehran Iran; ^6^ Cardiovascular Research Center Shahid Beheshti University of Medical Sciences Tehran Iran

**Keywords:** antiphospholipid syndrome, APS, massive pulmonary embolism, thrombocytopenia, thrombolytic therapy

## Abstract

Thrombocytopenia (TCP) is a well‐known contraindication for thrombolytic therapy due to the associated risk of bleeding. This report discusses a 34‐year‐old woman presented with massive pulmonary embolism (PE) and severe TCP. She was treated with systemic thrombolysis (ST), and fortunately, a good therapeutic response was achieved without significant bleeding. Further evaluations confirmed the diagnosis of antiphospholipid syndrome for the patient. This case highlights that ST can be a viable therapeutic option for managing massive PE in thrombocytopenic patients under specific circumstances, warranting further research into its safety and efficacy.

**Summary**

- Systemic thrombolysis may still be considered as a potentially effective and safe treatment option for massive pulmonary embolism in thrombocytopenic patients with antiphospholipid syndrome, even in the presence of relative contraindications.
- This antiphospholipid syndrome case highlights the potential safety and effectiveness of systemic thrombolysis, suggesting more research to assess such high‐risk situations and provide reliable protocols.

**Abbreviations**
aCL: anticardiolipin antibodies
aPL: antiphospholipid antibodies
APS: antiphospholipid syndrome
BP: blood pressure
HR: heart rate
IV: intra venous
PE: pulmonary embolism
PLT: platelet
RV: right ventricular
β2GPI: anti‐β2 glycoprotein‐I

## 1 Introduction

Massive pulmonary embolism (PE) is a challenging clinical issue that is defined as a massive thrombosis affecting more than 50% of pulmonary vessels. These patients are usually hemodynamically unstable and should be immediately diagnosed and treated [1]. The mortality rate is high, and most deaths occur within the first 6 h of the event due to multiple organ failure [2]. As a result, treatment of massive PE should be started immediately with systemic thrombolysis (ST), catheter‐directed thrombolysis, or surgical embolectomy [3]. Although ST may result in hemorrhagic complications [4], several former studies have suggested that it is associated with lower mortality rates and still remains the fundamental reperfusion therapy in these patients [5]. Patients with a higher risk of bleeding are preferred to be treated by catheter‐directed thrombolysis or surgical embolectomy. However, due to the lack of surgical and catheter‐based facilities and expert physicians in most medical centers [3], thrombolytic therapy in patients with contraindications for thrombolytic agents is still a challenging issue.

Antiphospholipid syndrome (APS), a prothrombotic autoimmune disorder, can complicate PE management due to concurrent hematologic abnormalities such as thrombocytopenia (TCP) [6, 7, 8]. Bleeding diathesis, such as TCP, is one of the contraindications of ST. Although TCP is considered a contraindication for thrombolytic therapy in ischemic stroke, the safety and efficacy of ST in thrombocytopenic PE patients are unknown [9]. In acute ischemic stroke, it is not necessary to await PLT count results before treatment with ST [10]. However, in the case of PE, there are only a few reports of using thrombolytic agents in patients with TCP [11, 12, 13]. We report a case of massive PE with severe TCP, which has been successfully treated with ST.

## 2 Case History/Examination

**FIGURE 1:**
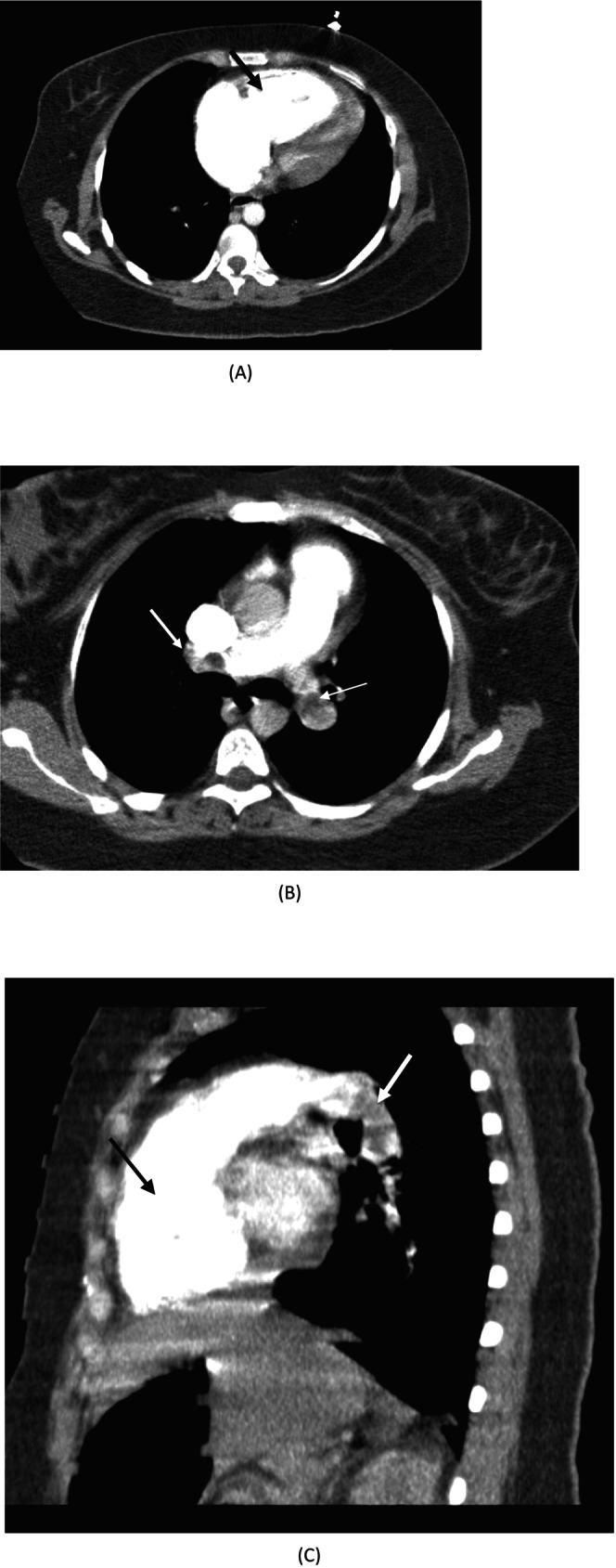
Chest CT angiography findings revealed right ventricular enlargement (black arrows in figures A&C) and pulmonary thromboembolism (white arrows in figures B&C). (A) Cross sectional view of the heart. (B) Cross sectional view of the pulmonary arteries. (C) Coronal view of the heart and pulmonary arteries.

A 34‐year‐old woman was referred to the emergency department with acute dyspnea and breathlessness 12 days after normal vaginal delivery (NVD). The patient was cyanotic and in respiratory distress. The patient was fully conscious, with a blood pressure (BP) of 86/60, a heart rate (HR) of 135 beats/min, and tachypnea with a respiratory rate (RR) of 40 breaths/min. On room air, she had an oxygen saturation of 70%. An electrocardiogram showed sinus tachycardia, a normal axis, an S_1_Q_3_T_3_ pattern, and T inversion in V1 to V4 leads. Bedside echocardiography revealed a normal LV size with preserved LV function (EF: 45%–50%), abnormal septal motion, severe right ventricular (RV) enlargement, moderate tricuspid regurgitation, and systolic pulmonary artery pressure of 50 mmHg. McConnell's sign was also seen. Chest computed tomography (CT) angiography confirmed the diagnosis of acute PE and represented filling defects in the left pulmonary artery and branches of the left and right lobar arteries (Figure 1), as well as evidence of RV strain. No abnormalities were found on abdominal, vaginal, or pelvic sonography. No evidence of active bleeding was observed on vaginal examination.

Furthermore, the patient's or her first‐degree relatives' medical records revealed no history of coagulation, rheumatologic, or thrombotic disorders. She had no history of arthritis, arthralgia, photosensitivity, skin rash, or mouth ulcers. Low PLT count had never been documented during pregnancy. The patient and first‐degree relatives had no history of genetic disorders. She claimed to have had an abortion when she was < 10 weeks pregnant 3 years ago. After the labor, she did not experience any abnormal bleeding. The patient had a PLT count of 180,000/μL at the time of delivery. The patient was not vaccinated for covid 19, and she did not have any suspicious symptoms of covid 19 infection.

## 3 Differential Diagnosis, Investigations, and Treatment

The patient was admitted with the diagnosis of massive PE and was initially treated with an infusion of alteplase 100 mg intravenously (IV) for 2 h. During alteplase infusion, symptoms disappeared. BP increased to 130/86 mmHg, HR decreased to 105/min, and O_2_ saturation increased to 92% in room air. After thrombolysis, subcutaneous enoxaparin 1 mg/kg (80 mg) twice daily was initiated. On‐arrival laboratory tests were received at the end of thrombolysis and revealed severe thrombocytopenia (PLT count: 48,000/μL), which falls below the commonly used threshold for defining severe thrombocytopenia in high‐risk procedural settings and immune‐mediated thrombocytopenia, as noted in international guidelines [14, 15]. Baseline laboratory tests are shown in Table 1. PLT count decreased gradually to 32,000/μL in the next 2 days. On the third day of hospitalization, a rheumatology service consultation was done for suspected antiphospholipid syndrome (APS) due to low PLTs and acute PE. Dexamethasone (8 mg IV every 8 h) was administered, and enoxaparin was maintained. Every day, the vital signs, headache, bleeding, and blurred vision were assessed. The patient's factor V Leiden G506A was normal, and triple assay tests, including Cardiolipin IgG, Cardiolipin IgM, Lupus Anticoagulant, Beta 2 glycoprotein IgG, and Beta 2 glycoprotein IgM, were positive. Increased thrombin time and Dilute Russell's Viper Venom Time (DRVVT) were found in the coagulation tests. The patient's hypercoagulability profile can be seen in Table 2 in detail.

**TABLE 1:** Patient's baseline laboratory tests.

Test	Result	Reference range
RBC count	4.4	4.2–5.4 million/μL
WBC^a^ count	19,900	4000–10,000/μL
Hemoglobin	13.5	11.6–15 mg/dL
Hematocrit	39	36%–46%
MCV	88	80–100 fL
MCH	29	27–33 pg
MCHC	33	32–36 g/dL
RDW	13	11.5%–14.5%
Platelet count	48,000	150,000–450,000/μL
Urea	31	6–24 mg/dL
Creatinine	1.7	0.7–1.4 mg/dL
AST^b^	57	NL < 37 IU/L
ALT^c^	30	NL < 41 IU/L
LDH^d^	772	140–280 U/L
Uric acid	4.6	4–8.5 mg/dL
PT^e^	13.2	11–13.5
INR^f^	1.3	< 1.1
ESR^g^	33	< 29 mm/h
CRP^h^	0.6	Neg < 0.6 mg/dL
TSH^i^	1.52	0.27–4.2 IU/mL
Troponin I	0.1	< 0.01

^a^ White blood cell.

^b^ Aspartate transferase.

^c^ Alanine aminotransferase.

^d^ Lactate dehydrogenase.

^e^ Prothrombin time.

^f^ International normalized ratio.

^g^ Erythrocyte sedimentation rate.

^h^ c‐Reactive protein.

^i^ Thyroid‐stimulating hormone.

**TABLE 2:** Patient's hypercoagulability profile.

Test	Result	Reference range
Factor V laden G506A	Normal	
Cardiolipin IgG	> 640	Neg < 12 Pl/mL
Cardiolipin IgM	30.7	Neg < 12 Pl/mL
Lupus anticoagulant	Positive	
Antiphospholipid Ab	41.69	Neg < 12 Pl/mL
Beta 2 glycoprotein IgG	155.1	NL < 20 U/mL
Beta 2 glycoprotein IgM	29.1	NL < 20 U/mL
Protein C	70.9	70–140
Protein S	69.9	60–140
C3	1.42	0.89–1.87 g/L
C4	0.20	0.1–0.4 g/L
CH50	120.4	41.2–95 U/mL
Thrombin time	33.1	15–19 s
DRVVT^a^	56.5	29–42 s

^a^ Dilute Russell's Viper Venom Time.

Since there was a lack of satisfactory clinical response to dexamethasone, intravenous immunoglobulin (IVIG) 20 g/day was started on the fifth day and continued for 5 days. After the first dose of IVIG, the PLT count increased to 81,000 and to 123,000 on the following days. On the sixth day, warfarin was started for the patient. Co‐administration of enoxaparin continued until the INR exceeded 2. Along with warfarin, hydroxychloroquine and aspirin were started.

## 4 Outcome and Follow‐Up

After 9 days, the patient was discharged in good general condition. No bleeding had occurred during hospitalization; the patient's condition was good and stable, and there were no new symptoms in regular follow‐ups. One month later, due to tapering of prednisolone and falling PLT counts, 1000 mg rituximab was infused into the patient, after which the PLT count became stable, and prednisolone was safely tapered to as low as 5 mg/day.

## 5 Discussion

Systemic thrombolytic therapy may induce several complications, including major hemorrhage. Bleeding is the most important complication and contraindication of thrombolytic therapy [9]. European Resuscitation Council (ERC) guidelines suggest that when no alternative treatments are available, the advantages of systemic thrombolytic therapy outweigh the disadvantages [16]. As a result, the physician should conduct a risk–benefit analysis before starting systemic fibrinolytic therapy for every individual patient [17].

Patients with PLT count < 100,000/mm^3^ are often excluded from ST efficacy assessment studies due to their bleeding diathesis, which is a contraindication for thrombolysis in patients with ischemic stroke [18, 19]. However, the safety and efficacy of thrombolysis in patients with acute PE and TCP are unknown [20]. On the other hand, it has been revealed that a PLT count of less than 50,000/μL is associated with a lower risk of venous thromboembolism, including PE, which suggests that the prevalence of TCP among PE patients is relatively low [21]. Moreover, normal PLT count is relatively low in some specific populations, which necessitates a lower cut‐off for fibrinolytic therapy [12]. Such pieces of evidence suggest that similar to the guidelines for ischemic stroke treatment, the physician does not have to await PLT count results during the acute management of massive PE [22, 23]. Although the prevalence of massive hemorrhage after thrombolytic therapy in PE is relatively low [24], the use of thrombolytic drugs in the treatment of PE has dramatically decreased due to fear of massive hemorrhage [25]. However, it has been reported that bleeding is mainly correlated with a history of bleeding rather than the PLT count [26].

Systemic thrombolytic therapy and even administration of anticoagulants among patients with antiphospholipid antibody syndrome (APS) and TCP is a matter of debate [27, 28], although concomitant thrombosis and severe TCP are extremely rare, and often occurred in rare cases of catastrophic APS [29]. Some studies have suggested TCP as a risk factor for thrombosis in APS patients [30, 31]. However, in this patient, massive PE occurred with severe TCP, and she was not a catastrophic APS.

There are only a few reports regarding successful systemic thrombolytic therapy of massive PE in thrombocytopenic patients in the literature [32, 33]. In this case, which presented with unstable hemodynamics, systemic thrombolytic therapy was initiated promptly after confirming the diagnosis of massive PE. Given the critical condition and absence of overt bleeding signs, and considering the unavailability of timely access to catheter‐based or surgical interventions, thrombolysis was deemed the most appropriate option, with careful monitoring for potential bleeding complications. If we knew about TCP before thrombolysis, half‐dose administration of alteplase may have been the acceptable alternative therapeutic approach [34]. However, treatment with full‐dose alteplase in this patient with a PLT count less than 100,000 did not result in any minor or major hemorrhage, while it has been reported that patients treated with alteplase had a higher rate of hemorrhage and a lower rate of recurrent PE versus anticoagulant therapy alone [35].

In this case, systemic corticosteroids (dexamethasone) were initiated upon suspicion of APS‐associated immune thrombocytopenia, a known hematologic manifestation of APS. However, due to a suboptimal platelet response, IVIG was added as second‐line therapy to promote a more rapid and sustained increase in platelet count. The use of corticosteroids and IVIG in APS is supported by existing literature, particularly in cases where thrombocytopenia is severe or refractory [36]. These immunomodulatory treatments are commonly used to mitigate the autoimmune‐mediated destruction of platelets and are essential for safely initiating or continuing anticoagulation in patients with high thrombotic risk.

In evaluating whether the patient had primary APS or APS secondary to a systemic autoimmune disorder such as systemic lupus erythematosus (SLE) or mixed connective tissue disease (MCTD), we performed a comprehensive clinical and laboratory assessment. The patient exhibited no clinical features suggestive of SLE or MCTD, such as malar rash, arthritis, mucosal ulcers, or photosensitivity. Laboratory investigations were also negative for antinuclear antibodies (ANA), anti‐dsDNA, ANCA, and the direct Coombs test, and there was no evidence of hemolysis. Given the presence of triple‐positive antiphospholipid antibodies and the absence of additional autoimmune markers or symptoms, this case is best classified as primary antiphospholipid syndrome. Recognizing this distinction is essential, as it may influence long‐term management and risk stratification.

Although this case report presented a successful systemic thrombolytic therapy of massive PE in a thrombocytopenic APS patient, further studies should be conducted to evaluate the efficacy and side effects of the thrombolytic agents in this condition. Future research should focus on prospective studies or case series evaluating the safety and efficacy of systemic thrombolysis in patients with thrombocytopenia, particularly those with APS. Additionally, the development of evidence‐based guidelines or risk stratification tools for managing high‐risk PE patients with contraindications to thrombolysis is warranted.

In conclusion, ST may be considered in select cases of massive PE with TCP due to APS, particularly when other treatment modalities are unavailable and the patient is hemodynamically unstable, but further studies will be needed to confirm our findings.

## Author Contributions

**Arman Ahmadzadeh:** conceptualization, methodology, supervision, writing – original draft, writing – review and editing. **Samad Nazar Poor:** data curation, formal analysis, methodology, validation, writing – original draft. **Reza Hamneshin Behbahani:** data curation, writing – original draft, writing – review and editing. **Pegah Soltani:** data curation, writing – original draft, writing – review and editing. **Arian Tavasol:** data curation, writing – original draft, writing – review and editing. **Mehdi Sheibani:** conceptualization, methodology, project administration, supervision, writing – review and editing.

## Consent

Written informed consent was obtained from the patient for publication of this case report and any accompanying images.

## Conflicts of Interest

The authors declare no conflicts of interest.

## Data Availability

The data supporting the findings of this case report are included within the article. Due to the nature of the study (individual case report), no additional datasets were generated or analyzed. Further details are available from the corresponding author upon reasonable request, while ensuring patient confidentiality.
